# Malnutrition-related symptom clusters and quality of life in nasopharyngeal carcinoma patients during radiotherapy: a network analysis

**DOI:** 10.1186/s12885-026-15694-z

**Published:** 2026-02-13

**Authors:** Meng-Yu Hao, Feng-Yan Li, Su-Man Zhang, Yu-Xian Yang, Yu-Xi Xiong, Hang-Yu Wang, Yao Zhuang Chuah, Zi-Hang Chen, Ling-Xin Xu, Peng Sun, Jian Ji, Lecheng Jia, Hua Li, Yanfei Liu, Ying Sun, Jia-Wei Lv, Yan Li, Guan-Qun Zhou

**Affiliations:** 1 Department of Radiation Oncology, State Key Laboratory of Oncology in South China, Guangdong Key Laboratory of Nasopharyngeal Carcinoma Diagnosis and Therapy, Guangdong Provincial Clinical Research Center for Cancer, Sun Yat-sen University Cancer Center, Guangzhou, 510060 P. R. China; 2 Zhongshan School of Medicine, Sun Yat-sen University, Guangzhou, 510060 China; 3 Department of Oncology, The Eighth Affiliated Hospital of Sun Yat-sen University, Shenzhen, China, People’s Republic; 4 School of Biomedical Engineering, Southern Medical University, Guangzhou, 510515 China; 5 Department of Medical Oncology, State Key Laboratory of Oncology in South China, Guangdong Provincial Clinical Research Center for Cancer, Sun Yat-sen University Cancer Center, Guangzhou, 510060 China; 6 Beijing Ansheng Xiangyuan Technology Development Co., Ltd, Guangzhou, 510030 China; 7 Shanghai United Imaging Healthcare Co., Ltd, Shanghai, 201807 China

**Keywords:** Nasopharyngeal carcinoma, Symptom clusters, Malnutrition, Quality of life, Network analysis, Radiotherapy

## Abstract

**Background:**

To identify symptom clusters (SCs) of nasopharyngeal carcinoma (NPC) patients during radiotherapy and examine the relative importance of specific symptoms in relation to quality of life (QoL).

**Methods:**

This cross-sectional study recruited non-metastatic NPC patients undergoing radiotherapy at Sun Yat-sen University Cancer Center (August 23—24, 2023). Acute toxicities, malnutrition, and QoL were assessed using the patient-reported outcome version of the Common Terminology Criteria for Adverse Events (PRO-CTCAE), modified Patient-Generated Subjective Global Assessment (mPG-SGA), and European Organization for Research and Treatment of Cancer Quality of Life Questionnaire Head and Neck 35 (EORTC QLQ-H&N35), respectively. Exploratory factor analysis and network analysis were used to identify SCs and characterize central and bridge symptoms. Multivariable logistic regression was conducted to explore factors associated with QoL.

**Results:**

A total of 437 eligible patients (73.46% male; median [IQR] age, 47 [38—55]) were included. Most patients reported ≥ 5 acute toxicities (88.56%) and severe malnutrition (75.06%). Three SCs (corresponding central symptoms) were identified from 18 prevalent items of PRO-CTCAE: general SC (anxiety), head-neck SC (dysphagia), and gastrointestinal SC (nausea). Malnutrition showed high bridge connectivity across SCs. The network remained relatively stable across early and late radiotherapy phases. Among multiple variables examined, the head–neck SC showed the strongest association with poorer QoL (β = 1.805, *P* < 0.001).

**Conclusions:**

NPC patients experience multiple co-occurring symptoms that organize into distinct clusters associated with QoL throughout radiotherapy. Dysphagia in head-neck SC and malnutrition deserve to be priorities for early management to relieve the global network burdens.

**Supplementary Information:**

The online version contains supplementary material available at 10.1186/s12885-026-15694-z.

## Introduction

Nasopharyngeal carcinoma (NPC) is a specific type of head and neck carcinoma (HNC) particularly prevalent in East and Southeast Asia, accounting for over 70% of global cases [1]. Although advancements in imaging modalities and intensity-modulated radiotherapy (RT) have contributed to the 5-year locoregional control rate exceeding 90% [2], many NPC patients continue to experience multiple treatment-related toxicities, malnutrition, and poor quality of life (QoL) during RT [3]. Previous studies have predominantly focused on single symptoms assessed by physicians [4, 5], while patient-reported outcomes (PROs), which more comprehensively capture patients’ subjective symptom burden, have been relatively underutilized, potentially limiting personalized supportive care [6].

Treatment-related symptoms in cancer patients rarely occur in isolation. Instead, they tend to co-occur and interact dynamically, forming symptom clusters (SCs) that may reflect shared underlying biological mechanisms, treatment-related tissue injury, or psychosocial processes [7]. Growing evidence suggests that such synergistic interactions among co-occurring symptoms may exert a greater detrimental impact on patient outcomes than the sum of individual symptoms [8]. Common reported SCs include gastrointestinal SC (e.g., nausea-vomiting-anorexia), psychological SC (e.g., anxiety-depression), and cancer-specific SC (e.g., cough-expectoration-hemoptysis in lung cancer) [9–11], yet NPC research remains scarce. A seminal cross-sectional study in 2017 identified four SCs in the late RT phase (weeks 4–7) [12] and highlighted the nutrition impact SC was the most severe SC, but it omitted symptom interactions and potentially escalating toxicity in the early RT phase.

Compared with single-symptom analyses, cluster-based approaches more closely reflect patients’ real-world experiences and may reveal cumulative and synergistic effects that are not apparent when symptoms are considered independently [8]. Network analysis, originally developed in social and psychological sciences, has recently emerged in oncology research [13]. Rather than viewing symptoms as passive indicators of a latent construct, network analysis conceptualizes symptoms as mutually interacting components within a complex system. This framework enables the identification of highly connected or bridging symptoms through centrality metrics (e.g., strength and betweenness), thereby highlighting nodes that may exert disproportionate influence on the overall symptom structure. Early recognition of highly connected or bridging nodes [14] may help prioritize targets for clinical assessment and hypothesis generation regarding supportive interventions.

Notably, malnutrition is highly prevalent in NPC patients undergoing radiotherapy and is closely intertwined with symptom burden. Treatment-related symptoms such as dysphagia, mucositis, taste alterations, and diarrhea can directly impair oral intake and nutrient absorption, contributing to weight loss and sarcopenia [15]. Conversely, malnutrition and muscle wasting may aggravate symptoms like fatigue, weakness, and reduced treatment tolerance, potentially forming a self-reinforcing cycle. Yet, few studies have examined malnutrition as a bridge linking different SCs within the symptom network.

QoL is a multidimensional concept encompassing physical, psychological, and social domains. Different toxicities and other factors may ultimately affect QoL in distinct ways [16, 17]. To date, however, no prior NPC study has systematically integrated SCs, nutritional status, and QoL within a unified network framework.

To address these gaps, we conducted this comprehensive cross-sectional study in an endemic region of NPC, incorporating various PRO questionnaires. The aims of this study were to (1) depict the current real-world burdens of acute toxicities during all RT phases and (2) determine SCs and their network relationships with malnutrition and QoL.

## Methods

### Design and participants

This cross-sectional study was conducted at the Yue Xiu campus of Sun Yat-sen University Cancer Center (SYSUCC), equipped with 18 linear accelerators and 65 treatment planning systems (TPS) between August 23 and 24, 2023. Deep learning-based auto-contouring of organs at risk (OARs) was routinely implemented to optimize planning efficiency [18]. Inclusion criteria were the following: age ≥ 18 years; pathologically confirmed primary non-metastatic NPC; undergoing RT at the Yue Xiu campus; Karnofsky performance scale score ≥ 70; freedom from mental illness. Exclusion criteria included pregnancy or lactation, prior head and neck surgery or RT, and pre-treatment imaging performed more than three months before enrollment. Eligible patients were notified of the survey location and time by text message 24 h in advance and reminded if absent. This study was approved by the Institutional Review Board of SYSUCC (approval number B2023-482-01). Written informed consent was obtained from all participating patients.

### Procedure and assessment

Demographic and clinical data were obtained from the big-data intelligence framework [19] of SYSUCC. Electronic questionnaires were administered via the Wenjuanxing online survey platform (wjx.cn) at the Radiation Center during patients’ RT waiting time. Physicians offered standardized face-to-face explanations as needed.

The patient-reported outcome version of the Common Terminology Criteria for Adverse Events (PRO-CTCAE), a validated instrument widely used in oncology [20, 21], was used to assess 18 pre-selected symptoms. These symptoms were chosen a priori based on their established clinical relevance [13] to NPC and radiotherapy-related toxicities, as well as sufficient prevalence [22] and variability during treatment to ensure analytical stability [23]. Importantly, symptom selection was predefined and not modified based on subsequent analytical results.

Exploratory factor analysis (EFA) was performed using principal axis factoring [23], which emphasizes shared variance among symptoms and is considered appropriate for identifying underlying symptom structures in exploratory settings. An oblique rotation (Oblimin) was applied to allow for correlations between symptom domains. The adequacy of factor analysis was confirmed by a Kaiser-Meyer-Olkin (KMO) index > 0.7 and a significant Bartlett’s sphericity test (*P* < 0.05) [22].

SCs were identified according to the following predefined criteria: a minimum of three symptoms per cluster [24], eigenvalues > 1, absolute factor loadings of items in SCs > 0.35, Cronbach’s α ≥ 0.70, and clinical relevance [11]. SC severity scores were calculated as the mean sum of all symptom scores within each cluster. No data-driven or post hoc modifications were applied to symptom selection, factor retention, loading thresholds, or clustering criteria based on analytical results.

Nutritional status was evaluated using the modified Patient-Generated Subjective Global Assessment (mPG-SGA), a validated and widely accepted nutritional screening tool for Chinese cancer populations [25]. Anthropometric data, including body weight and height in the preceding 6 months, were verified in the Hospital Information System. Percentage weight loss (WL), body mass index (BMI), and lean body mass (LBM) were calculated using established formulas [26, 27]. The modified weight loss grading system (WLGS) as reported previously [28] (Table S1) was used to precisely describe the WL. Body composition was assessed by measuring the skeletal muscle area (SMA) at the third cervical vertebral (C3) level on radiotherapy planning computed tomography images using ITK-SNAP software (version 4.0) [29]. C3-derived SMA values were converted to whole-body skeletal muscle estimates using validated conversion Eqs. [30, 31].

QoL was assessed using the European Organization for Research and Treatment of Cancer Quality of Life Questionnaire Head and Neck 35 (EORTC QLQ-H&N35) (Chinese version 1.0) [32, 33]. This questionnaire is specifically designed to evaluate symptom-specific QoL in HNC patients and is widely used in observational studies [34]. It comprises seven multi-item scales and 11 single-item symptom measures. Global QoL scores were linearly transformed as the mean sum of standardized scale scores [35]. Symptom severity and global QoL were categorized using established cutoff values: 0 (none), 1–33.33 (mild), 33.34–66.67 (moderate), and > 66.67 (severe) [16]. Moderate or severe global QoL was defined as “poor QoL”.

### Network analysis

An undirected network was constructed based on the identified SCs using the R package “qgraph”. Nodes represented individual symptoms or clinical variables, and edges represented adjusted associations between nodes, with thicker edges representing stronger associations. Spearman correlation analysis was applied for ordinal questionnaire options. To reduce spurious associations and enhance interpretability, the least absolute shrinkage and selection operator regularization was applied, combined with extended Bayesian information criterion model selection. A force-directed spring layout was performed to position nodes with the strongest correlations at the center, weaker connected nodes at the periphery, and nodes with similar characteristics closer to each other.

Centrality and bridge centrality indices were estimated using the R packages “qgraph” and “networktools” [14]. “Strength” (the absolute sum of a node’s direct connections) and “expected influence” (the arithmetic sum of a node’s direct connections) were standardized using z-score normalization [36]. The other centrality indices (“closeness” and “betweenness”) were not included due to their instability [8]. “Bridge strength” (the absolute sum of inter-cluster edge weights of a node) [37] was widely used to identify symptoms linking different SCs.

The stability of the centrality indices was evaluated using the bootstrap method in the “bootnet” R package (nBoots = 1000). The case-dropping bootstrap approach was employed to quantify the expected influence stability, and a correlation stability coefficient (CS-coefficient) greater than 0.25 was regarded as robust stability [38]. A network comparison test (NCT) was conducted using the “Network Comparison Test” R package [39] to examine differences between subgroup networks in terms of network structure, edge strength, and global strength. Considering the clinical value and balanced sample sizes, we selected the RT phases using median fractions as the cutoff value for the NCT.

### Statistical analysis

**Fig. 1:**
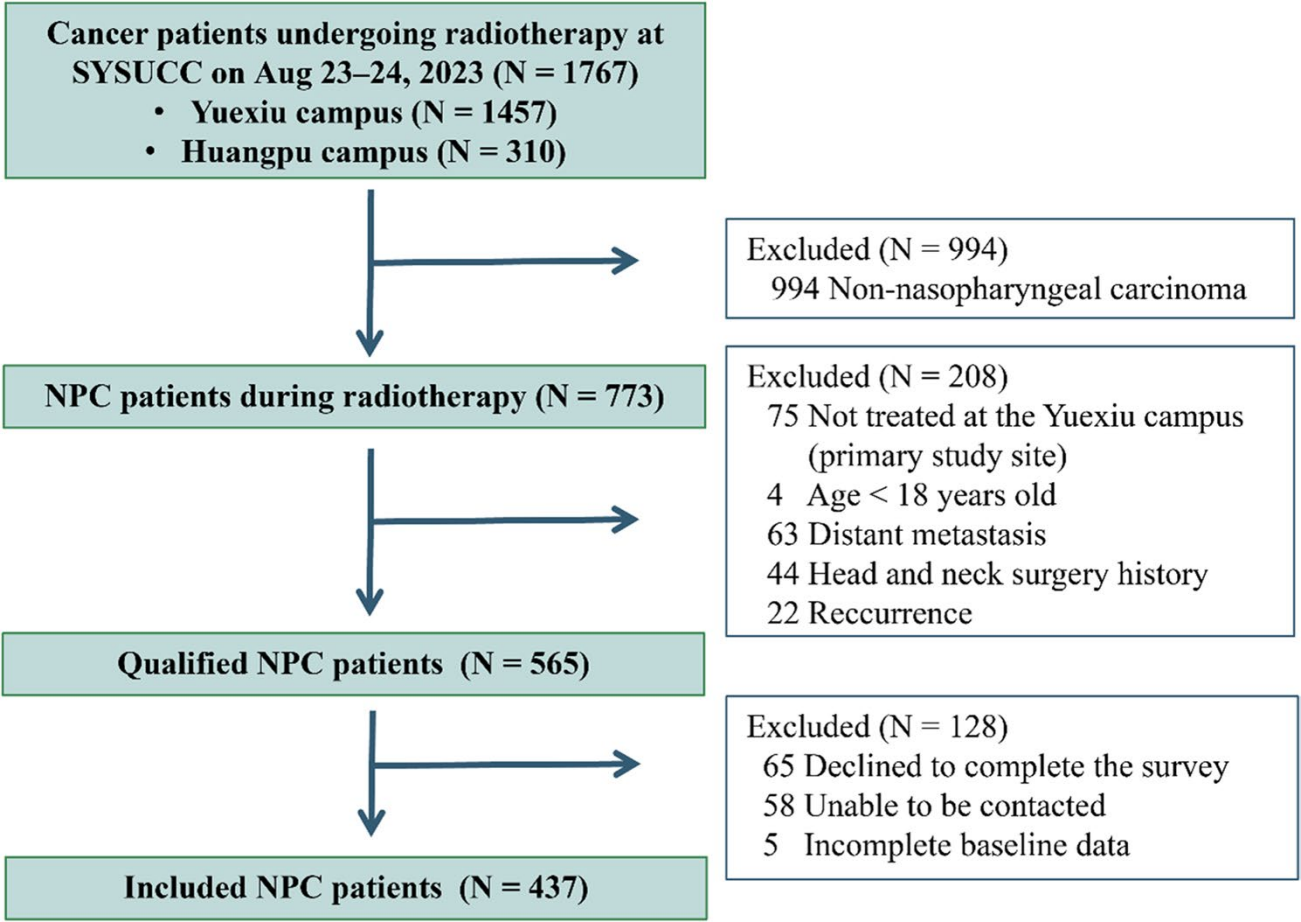
Flowchart of the cross-sectional study. SYSUCC, Sun Yat-sen University Cancer Center; NPC, nasopharyngeal carcinoma

We used convenience sampling from the entire NPC population receiving RT at SYSUCC without a pre-calculated sample size. Given the low proportion of missing data and the assumption of a missing completely at random mechanism [40], complete-case analysis (listwise deletion) was applied. Patients with incomplete records were excluded (Fig. 1).

All statistical analyses were performed using the IBM SPSS Statistics version 25.0 and R version 4.4.1. Continuous data were represented as mean ± standard deviation (SD) if normally distributed or as median ± interquartile range (IQR) if skewed. Categorical variables were summarized as frequency and percentages. Group comparisons were conducted using Mann-Whitney U or Kruskal-Wallis tests for continuous or ordinal variables, and the Chi-square or Fisher’s exact tests for categorical variables. Spearman rank correlation coefficients (*r*) were calculated to assess the correlation.

Given the exploratory and hypothesis-generating nature of this study, no formal adjustment for multiple comparisons was applied. All *P* values are therefore reported as descriptive measures rather than confirmatory statistical inference. Factors that were significant in the univariate analysis or clinically valuable were selected for further multivariate logistic regression analysis of binary QoL using the forward selection method. A two-tailed *P* < 0.05 was considered to be statistically significant.

## Results

### Study workflow and patient characteristics

Between August 23 and 24, 2023, 1767 cancer patients underwent RT at SYSUCC (Table S2). Among them, 773 (43.75%) were NPC patients who were managed in the MOSAIQ integration platform [41]. Through decision tree screening in our NPC-specific database, 565 patients met the predefined inclusion criteria and were consecutively recruited via message alerts (Fig. 1). Following the two-day field investigation, 437 patients completed the questionnaires and were included in the final analysis (Table 1, S3). The cohort comprised predominantly males (73.46%) with a median age of 47 years (IQR: 38–55). All of them were receiving radical RT (76.4% prescribed a radiation dose of 6996 cGy in 33 fractions), and 78.95% had undergone induction chemotherapy before (Table S4).

**Table 1:** Scores of PRO-CTCAE and mPG-SGA items (*n* = 437)

PRO-CTCAE items	*N* (%)	mPG-SGA items	*N* (%)
Xerostomia	394 (90.16)	Food intake	
Grade 3–4	112 (25.63)	Unchanged or more	34 (7.78)
Decreased appetite	388 (88.79)	Normal food but eating less	95 (21.74)
Grade 3–4	130 (29.75)	Little solid food	196 (44.85)
Taste alterations	375 (85.81)	Only liquids	71 (16.25)
Grade 3–4	168 (38.44)	Only nutritional supplements	15 (3.43)
Fatigue	364 (83.30)	Only tube feedings/nutrition by vein	1 (0.23)
Grade 3–4	55 (12.59)	Very little of anything	25 (5.72)
Nausea	359 (82.15)	Nutrition impact symptoms (NIS)	
Grade 3–4	92 (21.05)	Taste disorder (1 point)	235 (53.78)
Mouth/throat sores	321 (73.46)	Dry mouth (1 point)	200 (45.77)
Grade 3–4	88 (20.14)	Nausea (1 point)	192 (43.94)
Dysphagia	319 (73.00)	No appetite (3 points)	183 (41.88)
Grade 3–4	85 (19.45)	Swallowing problems (2 points)	177 (40.5)
Vomiting	293 (67.05)	Vomiting (3 points)	98 (22.43)
Grade 3–4	61 (13.96)	Smell disorder (1 point)	80 (18.31)
Immobility	275 (62.93)	Constipation (1 point)	74 (16.93)
Grade 3–4	55 (12.59)	Feel full quickly (1 point)	70 (16.02)
Constipation	272 (62.24)	Pain (3 points)	57 (13.04)
Grade 3–4	62 (14.19)	Diarrhea (3 points)	9 (2.06)
Insomnia	260 (59.5)	Activities and function	
Grade 3–4	29 (6.64)	Normal with no limitations	162 (37.07)
Anxiety	244 (55.84)	Fairly normal with mild limitations	163 (37.3)
Grade 3–4	20 (4.58)	Abnormal with limitations < half day	57 (13.04)
Dizziness	236 (54.00)	Little activity and rarely out of bed	55 (12.59)
Grade 3–4	21 (4.81)	Weight loss	
Radiation skin reaction	227 (51.95)	In the past six months, Mean (SD)	7.22 (6.52)
Grade 3–4	16 (3.66)	In the recent two weeks, *N* (%)	363 (83.06)
Cough	219 (50.11)	Age	
Grade 3–4	16 (3.66)	> 65 years	25 (5.72)
Palpitations	172 (39.36)	≤ 65 years	412 (94.28)
Grade 3–4	6 (1.37)	mPG-SGA total scores, Mean (SD)	10.60 (5.48)
Abdominal pain	147 (33.64)	Well-nourished (0 point)	6 (1.37)
Grade 3–4	10 (2.29)	Mildly malnourished (1–2 points)	24 (5.49)
Shortness of breath	129 (29.52)	Moderately malnourished (3–6 points)	79 (18.08)
Grade 3–4	5 (1.14)	Severely malnourished (≥ 7 points)	328 (75.06)

*PRO-CTCAE,* Patient-reported outcome version of the Common Terminology Criteria for Adverse Events; *mPG-SGA*, Modified Patient-Generated Subjective Global Assessment; *N*, number of patients; *SD*, Standard deviation

### Symptom burdens and nutritional impact

Overall, 387 patients (88.56%) experienced at least five acute symptoms assessed by PRO-CTCAE concurrently during RT (Table 1). Xerostomia was the most prevalent (90.16%), while taste alterations showed the highest severity (38.44% Grade 3–4). According to mPG-SGA, only 8.24% of patients reported no nutritional impact symptoms, and 75.06% were classified as having severe malnutrition. Based on body composition analysis, sarcopenia was identified in 171 patients (39.13%).

Within the EORTC QLQ-H&N35 questionnaire, WL had the highest score, followed by the nutritional supplements scale (Table S3). In the past week preceding the survey, 356 patients (81.46%) experienced recent WL, while 15.79% stated no change. In the past six months, 278 patients (63.62%) experienced WL ≥ 5%, with 135 of them losing ≥ 10%. Additionally, patients exhibited a wide variation in BMI (13.2–33.5 kg/m^2^). After BMI adjustment, 56.29% of patients experienced grade 3–4 WL.

### Symptom clusters and specific roles

Through the EFA (KMO index = 0.91, *P* < 0.001 in Bartlett’s test), three factors with eigenvalues > 1 were extracted, explaining 56.32% of the variance with a Cronbach’s alpha of 0.863. Based on symptom composition and clinical relevance, these three SCs were labelled as the general SC, head-neck SC, and gastrointestinal SC, with detailed compositions shown in Table 2.

**Table 2:** The results of exploratory factor analysis

Symptom cluster	Symptoms	Factor loading		
		1	2	3
Cluster 1: General symptom cluster (M = 0.76, SD = 0.58)	Anxiety	0.795*	0.315	-0.360
	Shortness of breath	0.758*	0.294	-0.284
	Palpitations	0.749*	0.313	-0.349
	Dizziness	0.711*	0.299	-0.464
	Fatigue	0.699*	0.436	-0.520
	Insomnia	0.616*	0.364	-0.192
	Abdominal pain	0.624*	0.362	-0.237
	Cough	0.619*	0.502	-0.173
	Immobility	0.541*	0.435	-0.441
Cluster 2: Head-neck symptom cluster (M = 1.50, SD = 0.85)	Dysphagia	0.482	0.853*	-0.259
	Mouth/throat sores	0.437	0.798*	-0.217
	Taste changes	0.220	0.712*	-0.228
	Radiation skin reaction	0.538	0.649*	-0.031
	Xerostomia	0.413	0.687*	-0.448
Cluster 3: Gastrointestinal symptom cluster (M = 1.45, SD = 0.90)	Nausea	0.525	0.380	-0.893*
	Vomiting	0.529	0.398	-0.839*
	Decreased appetite	0.461	0.638	-0.669*
	Constipation	0.399	0.512	-0.411*
Initial Eigenvalues		7.412	1.572	1.153
Cumulative %		41.176	49.912	56.32
Cronbach’s α		0.863	0.810	0.808

Extraction method: principal axis factoring. Rotation method: oblimin with Kaiser normalization

*M,* Mean; *SD*, Standard deviation

*Loadings for the factor indicated

**Fig. 2:**
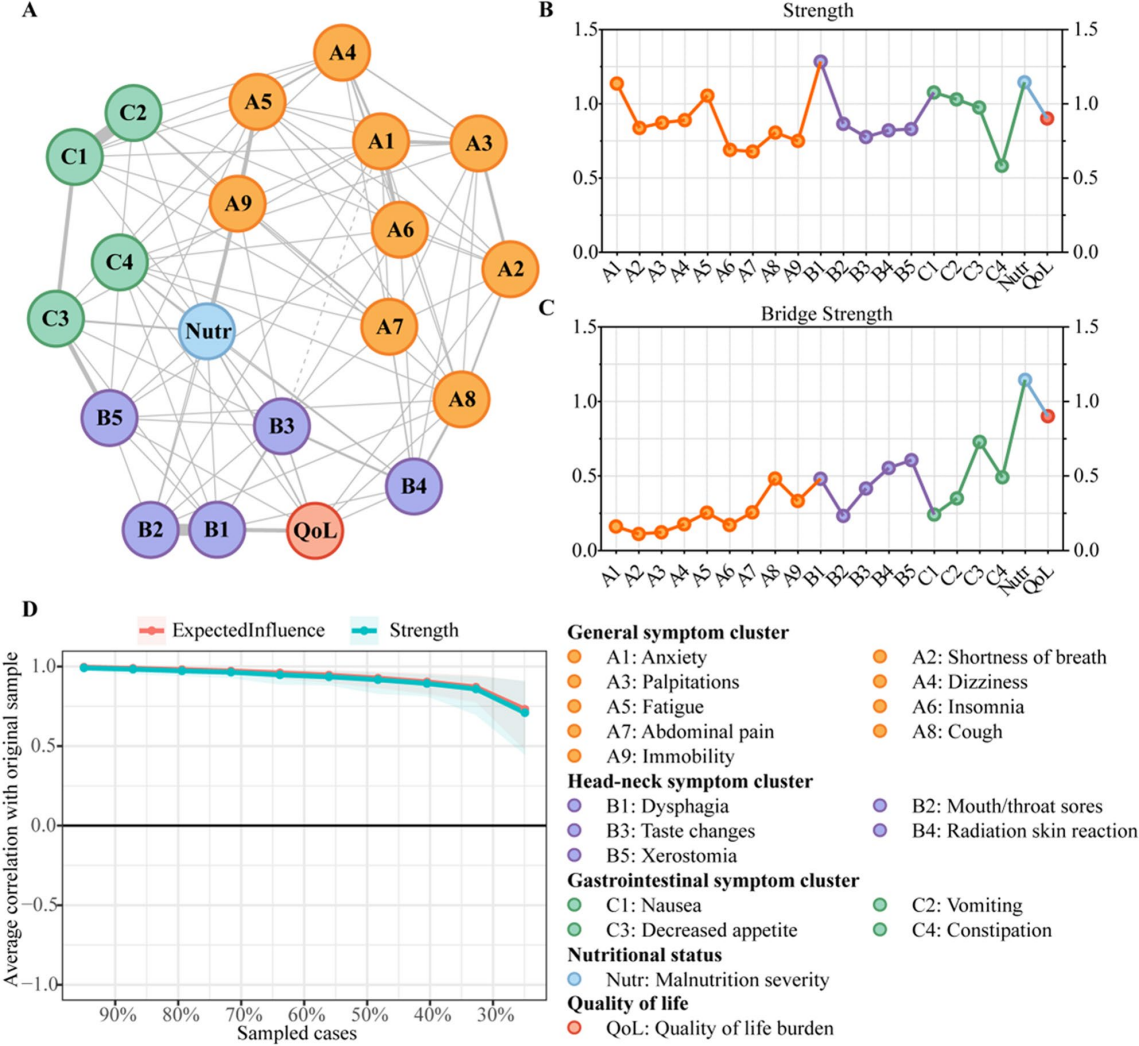
Network analysis of symptoms, malnutrition, and quality of life. **A** Network model with nodes corresponding to symptoms. Solid grey lines denote positive correlations, and dashed lines represent negative correlations. The length and thickness of the edges signify correlation strength. **B**-**C** Centrality indices and bridging indices of nodes in the network. The x-axis marks the nodes of the network. The y-axis shows the z-score normalized value of these indices. Dot colors align with network (A)’s scheme. **D** Centrality index stability evaluated by case-dropping subset bootstrap. The x-axis represents the percentage of original cases sampled per step. The y-axis represents the correlation between original and post-bootstrapping re-estimated networks’ centrality indices. Lines plot the estimated correlation coefficients at each step, with shaded areas denoting 95% confidence intervals

Network visualization revealed substantial interactions among symptoms. The strongest positive pairwise association was observed between nausea and vomiting (edge weight = 0.60), followed by mouth/throat sores and dysphagia (edge weight = 0.57; Table S5). Head-neck SC was positioned closer to the QoL node than the general or gastrointestinal SCs (Fig. 2A).

In the global network, dysphagia showed the highest strength, while malnutrition exhibited the strongest overall connectivity with other symptoms across different SCs (Fig. 2B-C). All PRO-CTCAE items were positively correlated with malnutrition scores (*r* = 0.31–0.57, *P* < 0.05; Table S6). At the subcluster level, anxiety, dysphagia, and nausea were central symptoms of general SC, head-neck SC, and gastrointestinal SC, respectively. The CS-coefficient was 0.673 in the bootstrap analysis (Fig. 2D). After adjustment for covariates associated with QoL in the univariate analysis (Table S4), the estimated network structure, strength indices (Fig. S1), and relative weight edges (Table S7) remained largely unchanged.

### Dominant factors associated with overall QoL

**Fig. 3:**
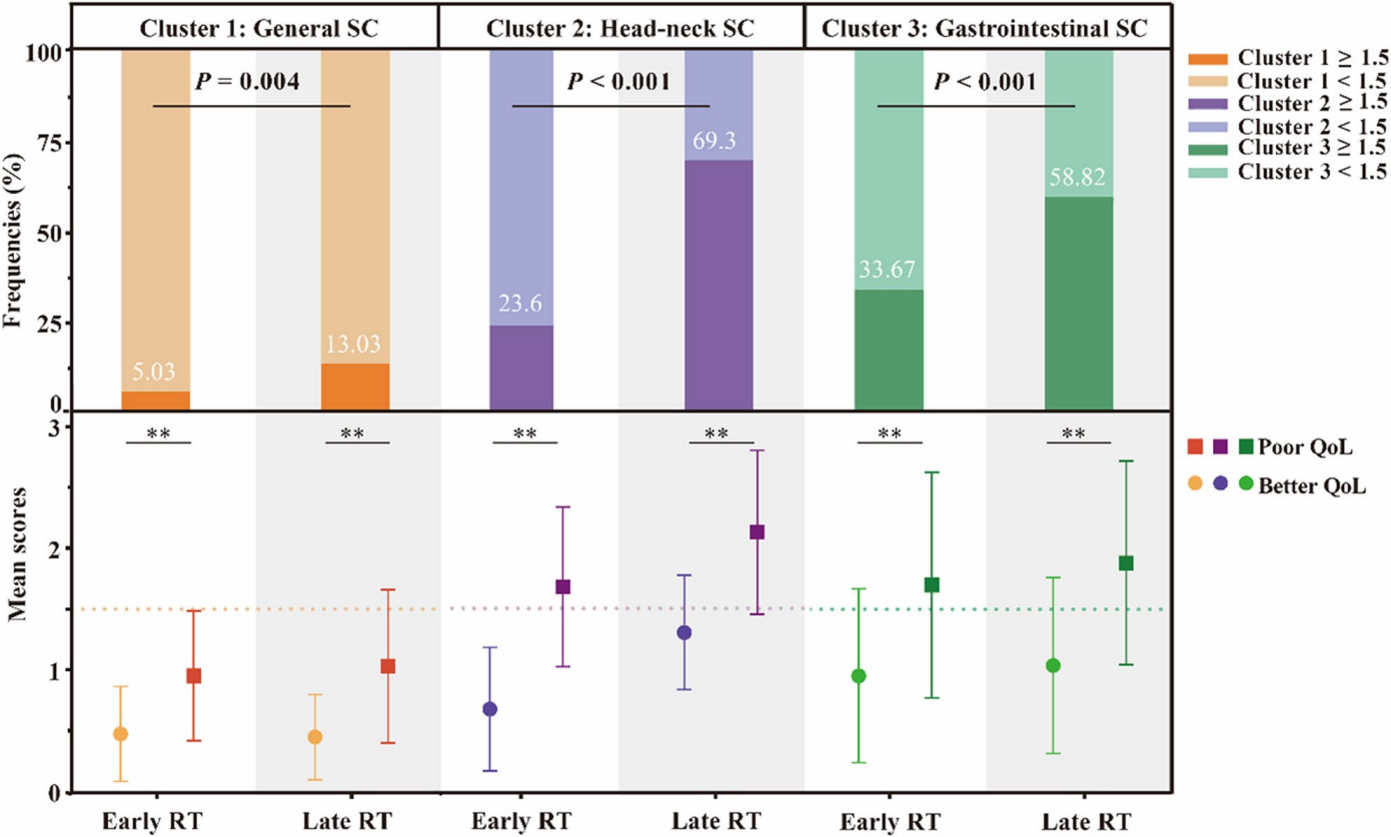
Prevalence and severity of symptom clusters of QoL subgroups in the early and late phases. SCs, symptom clusters; RT, radiotherapy; Early: < 18 fractions; Late: ≥ 18 fractions; QoL, quality of life; ** for *P* < 0.01; Data are expressed as mean ± standard deviation in the plot

The cohort exhibited marked overall QoL impairment (mean standardized score 35.7 ± 14.67). Univariate analysis revealed several vulnerable factors associated with poor QoL, including female gender, detectable EBV DNA before RT, high cumulative fraction and dosage, absence of replanning, severe modified WL in the past 6 months, and severe malnutrition (*P* < 0.05; Table S4). Patients with higher severity in all three SCs reported significantly poorer QoL scores (Fig. 3).

**Fig. 4:**
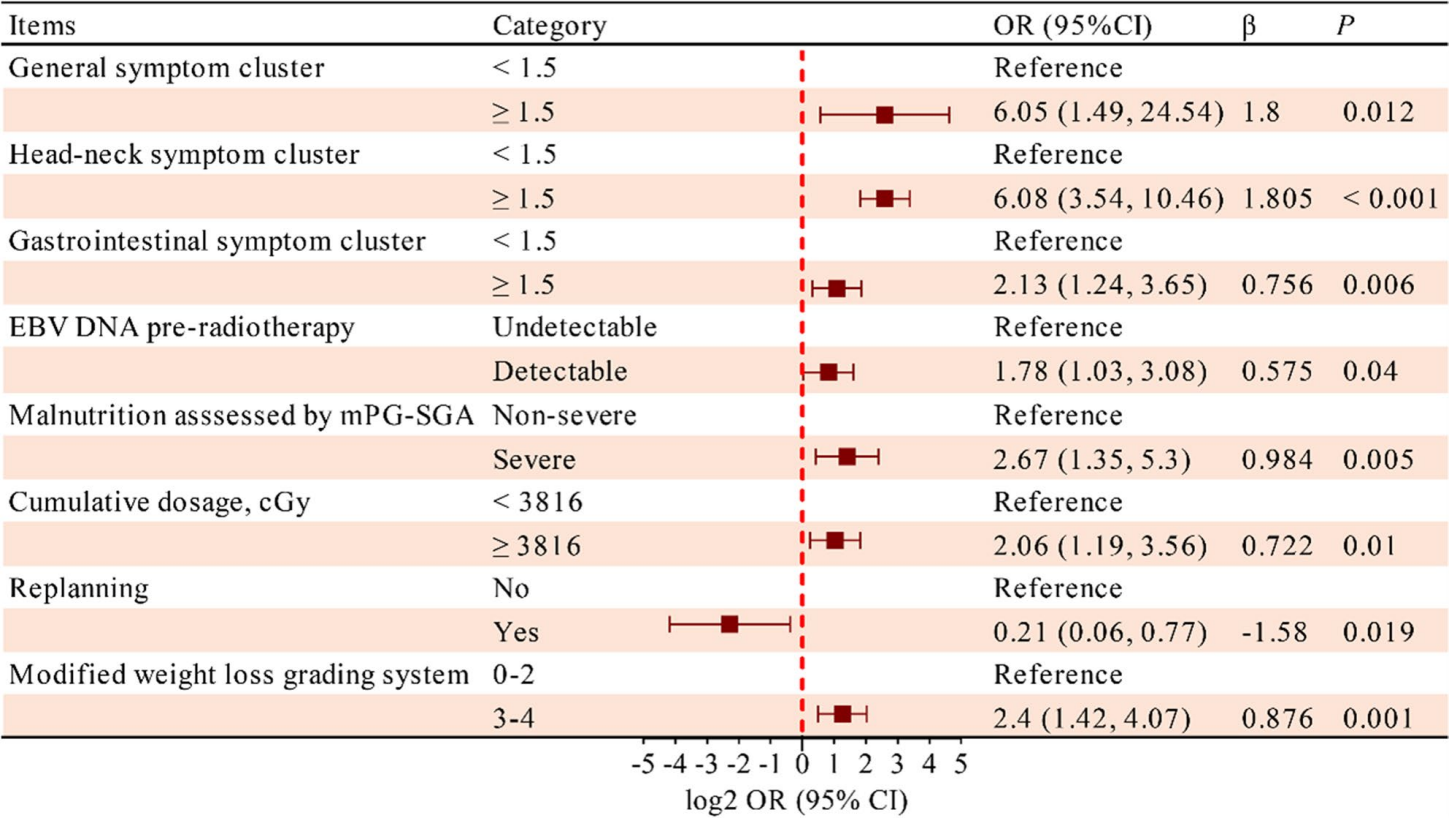
Multivariate logistic regression analysis of quality of life. EBV, Epstein-Barr virus; DNA, deoxyribonucleic acid; mPG-SGA, modified Patient-Generated Subjective Global Assessment; cGy, centiGray; OR, odds ratio; CI, confidence interval

In the multivariate logistic regression analysis, explanatory variables of binary overall QoL were identified. All three SCs, mPG-SGA grades, RT cumulative dosage, replanning, pre-RT EBV DNA detectable, and modified WL% (mWLGS) were retained as independent variables after adjustment for age, gender, TNM stage [42], and other covariates (R^2^ = 0.539, *P* = 0.04; Fig. 4). Significant positive correlations were observed among most single symptoms between EORTC QLQ-H&N35 and PRO-CTCAE (*r*_max_ = 0.77, *P* < 0.001; Fig. S2). Head-neck SC maintained the superior explanatory power over any individual symptom (β = 1.642, *P* < 0.001; Table S7).

### Subgroup analysis of different RT phases

Across radiotherapy weeks 1 to 7, most symptom-specific QoL scores exhibited a progressive worsening trend (*P* < 0.05), except for the nutritional supplements and feeding tube scales, which did not show clear temporal patterns (*P* = 0.079, *P* = 0.071; Fig. S3). For subgroup analyses, patients were stratified into early and late radiotherapy phases using the median number of fractions as the cutoff.

From the early (< 18 Fr) to the late RT phase (≥ 18 Fr), head-neck SC worsened sharply, while general SC showed relatively minor changes. The most prevalent and severe symptom shifted from xerostomia to taste disturbances (Fig. S4). EAF indicated slight differences in the prominent factor loadings of specific RT phases compared with those derived from the entire RT course (Table S9). However, central and bridge symptoms remained consistent across phases (Fig. S5), and NCT confirmed there was no significant difference in network strength and structure between phase subgroups (Fig. S6).

For the multivariate logistic regression analysis, in the early RT phase, general SC and malnutrition showed the highest β to explain the QoL status (R^2^ = 0.595, *P* < 0.001; Fig. S7). Pre-RT EBV DNA and detailed RT fractions were also indicative, but both lost significance in the late phase after adjustments. Head-neck SC remained independently associated with poorer QoL across both the early and late RT phases (β_early_ = 1.445, β_late_ = 1.576, *P* < 0.001). Gastrointestinal SC and modified WL emerged as critical factors associated with QoL (β_SC_ = 1.439, β_WL_ = 1.054, R^2^ = 0.368, *P* < 0.001) only in the late RT phase.

## Discussion

To the best of our knowledge, this cross-sectional study is the first to systematically analyze the relational network among SCs, malnutrition, and QoL based on NPC patient-reported outcomes across all RT phases in a high-incidence area. Malnutrition emerged as a prominent node and a bridge symptom linking other SCs. Head-neck SC, particularly dysphagia, showed high centrality and close associations with QoL impairment, highlighting potential priorities for symptom-focused supportive care.

Our findings add to the contemporary understanding of daily acute toxicity burdens in the era of precision RT. NPC patients experienced a wide spectrum of symptoms during RT in the real world. Although the overall severity of several symptoms appeared lower than previously reported, the symptom burden remained concerning (Table S10). WL, taste alterations and malnutrition were the most severe symptoms, whereas xerostomia exhibited the highest prevalence, pointing out unmet care needs. Notably, the symptom burden captured through PROs exceeded physician-assessed toxicities reported in prior clinical trials using CTCAE criteria [20, 43]. Given that PRO monitoring has been associated with improved QoL in prior studies [44], it deserves greater attention in daily clinical practice and research [45].

Malnutrition and symptoms were closely correlated and jointly associated with QoL. Unlike prior studies that uniformly grouped food-intake-related symptoms into nutritional impact SCs [46, 47], we evaluated malnutrition as an independent node using the modified Patient-Generated Subjective Global Assessment (mPG-SGA), the current gold standard for nutritional assessment [15]. The observed high bridge strength and central positioning of malnutrition across RT phases (Fig. S5) suggest its potential role in connecting multiple symptom clusters and its close association with poorer QoL. Multivariate analyses further demonstrated phase-dependent associations between malnutrition and QoL, with the strongest associations observed during the early RT phase (Fig. S5C, S7). These findings raise the hypothesis that early nutritional support may be an important component of supportive care, although causal relationships require confirmation in prospective and interventional studies.

Symptoms within SCs demonstrated heterogeneous but potentially synergistic associations with QoL. Head–neck SC as a whole explained a greater proportion of QoL variance than individual symptoms, suggesting potential synergistic interactions (Table S8). These patterns were reflected in the network structure, where dysphagia and mouth or throat sores exhibited the second strongest edge weights within the head–neck SC. Moreover, the head–neck SC showed the shortest path lengths and strongest connections to the QoL node (Fig. 2A). In contrast, the general SC exerted relatively weaker associations with QoL, compared with certain isolated symptoms, including shortness of breath and cough (Table S8), likely due to their lower node strengths (Fig. 2B) and looser internal connectivity (mean edge weight: 0.16 vs. 0.37 within the head-neck SC). Overall, these results provide a quantitative description of the associations between acute toxicity-related SCs and QoL in NPC and may help inform considerations for OAR sparing strategies [17].

QoL is a subjective and complex concept influenced by multiple factors beyond treatment-related toxicities. In our cohort, females reported more symptoms than males, which may relate to biological, hormonal, or sociocultural factors [48]. Detectable EBV DNA before RT also showed an independent association with poorer QoL. Copy numbers of EBV DNA released from NPC cells may reflect tumor burden and treatment response [49], thus potentially influencing QoL directly and indirectly [50]. Furthermore, worsening QoL correlates with increasing BMI-adjusted WL grades, especially in the late RT phase, suggesting that early identification of WL may be relevant for recognizing patients at risk of poorer QoL [51].

Different RT phases may require tailored supportive care strategies. Our phase-stratified analyses revealed potential dynamic symptom patterns over the course of treatment. In the early RT phase, gastrointestinal SC showed notable associations with QoL impairment, potentially related to induction chemotherapy exposure. Meanwhile, general SC, despite being the least severe, was also associated with QoL deterioration (β = 2.593, *P* = 0.01; Fig. S7A). Consistent with previous research [16], most QLQ-H&N35 domains worsened rapidly in weeks 2–3 and peaked at the end of RT, except for nutritional supplements use and feeding tube scales with low prevalence (Fig. S3). In the late RT phase, head-neck SC became the most severe and was strongly associated with damaged QoL, underscoring the need for intensified supportive care during this period.

Notably, replanning was associated with timely improvements in QoL during RT (β = -1.434, *P* = 0.03; Fig. S7B), extending previous observations regarding its long-term post-RT benefits [52, 53]. However, this observation should be interpreted cautiously, as only a small subset of patients underwent replanning, all during the late RT phase (Table S11). Whether earlier replanning may improve QoL warrants further investigation. In this context, our team has initiated recruitment for the first phase III clinical trial of artificial intelligence (AI)-assisted online adaptive RT for NPC (ChiCTR2400079473; NCT06516133), integrating longitudinal real-time PROs as one of the key endpoints. Our long-term goal is to explore more precise and intelligent strategies aimed at reducing treatment-related toxicities and improving QoL while maintaining oncologic outcomes.

This study has several notable strengths. First, it captured real-world PROs across the entire course of RT in a large endemic NPC cohort. Second, the integration of exploratory factor analysis and network analysis enabled simultaneous identification of SCs and their interaction patterns with malnutrition and QoL. Third, the findings are clinically interpretable and readily applicable to routine supportive care, enhancing their potential generalizability.

Several limitations should be acknowledged. First, patients with extremely severe toxicities may have been less likely to participate, potentially leading to the underestimation of symptom prevalence and severity. To minimize this bias, clinical staff reminded absent patients twice via text messages. Second, PROs may be subject to recall and reporting biases. Nevertheless, QoL is inherently subjective, and PROs remain an essential and validated approach for capturing patients’ symptom experiences. Third, symptom clustering was limited to a predefined subset of PRO-CTCAE items due to questionnaire compatibility and to minimize patient burden during active radiotherapy. Future studies may incorporate a broader range of physical, psychological, and social symptoms, or integrate complementary instruments, to further refine and expand symptom network analyses. Fourth, the cross-sectional design captures patients at different treatment trajectories rather than longitudinal symptom evolution, precluding causal inference. Finally, no formal correction for multiple testing was applied due to the exploratory nature of the analyses, which may increase the risk of type I error. Accordingly, the findings should be interpreted with appropriate statistical caution. Despite these limitations, the relatively large sample size allows depiction of average symptom distributions at specific RT phases and provides a foundation for future prospective studies.

Taken together, these findings should be interpreted within the exploratory scope of the study and primarily serve to generate hypotheses for future longitudinal and interventional research. Network centrality and bridge metrics indicate relative importance within the observed symptom structure rather than causal relationships. Nevertheless, this integrated cluster–network framework provides a complementary perspective on symptom assessment and may help inform more targeted and patient-centered supportive care strategies in NPC.

## Conclusions

NPC patients experience diverse co-occurring symptoms that organize into distinct clusters associated with QoL during RT. Dysphagia within the head–neck SC and malnutrition appear to be prominent features within the network and may represent important considerations for symptom assessment and supportive care, warranting further longitudinal investigation.

## Supplementary Information

Supplementary Material 1

## Data Availability

Research data are stored in an institutional repository and will be shared upon request to the corresponding author (zhougq@sysucc.org.cn).

## Abbreviations

NPC: Nasopharyngeal carcinoma
HNC: Head and neck carcinoma
RT: Radiotherapy
PROs: Patient-reported outcomes
QoL: Quality of life
SC: Symptom cluster
CTCAE: Common Terminology Criteria for Adverse Events
EORTC QLQ-H&N35: European Organization for Research and Treatment of Cancer Quality of Life Questionnaire Head and Neck 35
SYSUCC: Sun Yat-sen University Cancer Center
AJCC: American Joint Committee on Cancer
EBV: Epstein-Barr virus
DNA: Deoxyribonucleic acid
IQR: Interquartile range
SD: Standard deviation
TPS: Treatment planning systems
OARs: Organs at risk
EFA: Exploratory factor analysis
KMO: Kaiser-Meyer-Olkin
WL: Weight loss
BMI: Body mass index
mWLGS: Modified weight loss grading system
mPG-SGA: Modified Patient-Generated Subjective Global Assessment
CT: Computed tomography
SMA: Skeletal muscular area
LBM: Lean body mass
CS-coefficient: Correlation stability coefficient
NCT: Network comparison test
cGy: CentiGray
