# Supplementary material for: Malnutrition-related symptom clusters and quality of life in nasopharyngeal carcinoma patients during radiotherapy: a network analysis
Source: BMC Cancer. 2026 Feb 13;26:431. doi: 10.1186/s12885-026-15694-z (PMC13041334; doi:10.1186/s12885-026-15694-z)
Supplement: Supplementary file 1 — Supplementary Material 1 [file 12885_2026_15694_MOESM1_ESM.pdf]

Supplementary Materials

Table S1 Modified weight loss grading system 2
Table S2 Profiles of 1767 patients treated at SYSUCC 3
Table S3 Scores of EORTC QLQ-H&N35 (*n* = 437) 4
Table S4 Baseline characteristics of patients across different quality of life groups 5
Table S5 Weight of each connection in the GLASSO network without clinical covariates 7
Table S6 Spearman correlation coefficient of PRO-CTCAE and malnutrition 8
Table S7 Weight of each connection in the GLASSO network with clinical covariates9
Table S8 Multivariate logistic regression analysis of quality of life adding symptoms as continuous variables 10
Table S9 Exploratory factor analysis in the early and late radiotherapy phases 11
Table S10 Prevalence of acute symptoms in the previous study compared with our survey 12
Table S11 Radiotherapy fractions of patients who had received replanning 13
Fig.S1 Visual representation of the network after controlling for covariates 14
Fig.S2 Correlation matrix (Spearman, r) of the PRO-CTCAE and EORTC QLQ-H&N35 items 15
Fig.S3 Mean scores of EORTC QLQ-H&N35 scales during weeks 1-7 of radiotherapy16
Fig.S4 Symptom severity distribution of PRO-CTCAE in the early and late radiotherapy phases 17
Fig.S5 Estimated network model for the association between symptom clusters, malnutrition, and QoL in the early and late radiotherapy phases 18
Fig.S6 Network strength invariance test and structure invariance test 19
Fig.S7 Multivariate logistic regression analysis of quality of life in the early and late radiotherapy phases 20

Table S1 Modified weight loss grading system

Weight loss (%)	Body mass index (kg/m^2)				
	≥ 28	25—27.9	22—24.9	20—21.9	< 20
± 2.4	0	1	1	2	2
2.5—5.9	0	2	2	3	3
6—10.9	1	2	3	3	4
11—14.9	2	3	3	3	4
≥ 15	3	4	4	4	4

1. Xie H, Ruan G, Wei L, et al. Development and applicability of modified weight loss grading system in cancer: a real-world cohort study. Journal of cachexia, sarcopenia and muscle. Oct 2023;14(5):2090-2097. doi:10.1002/jcsm.13287

Table S2 Profiles of 1767 patients treated at SYSUCC

Variable	N (%)
**Primary cancer site**	
Head and neck cancer	925 (52.35%)
Nasopharynx	773 (43.75%)
Oral cavity	42 (2.38%)
Hypopharynx	23 (1.3%)
Larynx	22 (1.25%)
Salivary gland	21 (1.19%)
Oropharynx	19 (1.08%)
Nasal cavity or middle ear	10 (0.57%)
Sinus	6 (0.34%)
Unknown or other	9 (0.51%)
Non-head and neck cancer	842 (47.65%)
Digestive system tumor	201 (11.38%)
Breast cancer	197 (11.15%)
Female reproductive tumors	129 (7.3%)
Thoracic tumor	110 (6.23%)
Central nervous system tumor	76 (4.3%)
Urinary system tumor	53 (3%)
Hematological malignancies	31 (1.75%)
Soft tissue tumor	13 (0.74%)
Bone tumor	9 (0.51%)
Ocular tumor	8 (0.45%)
Skin tumor (melanoma)	6 (0.34%)
Retroperitoneal tumor	3 (0.17%)
Thyroid tumor	2 (0.11%)
Adrenal gland tumor	2 (0.11%)
Unknown or other	2 (0.11%)
**Treatment technique**	
VMAT	1160 (65.65%)
IMRT	394 (22.3%)
SBRT	116 (6.56%)
TOMO	72 (4.07%)
3D-CRT	25 (1.41%)
**Treatment planning system**	
Eclipse	859 (48.61%)
Monaco	715 (40.46%)
United imaging	118 (6.68%)
TOMO	75 (4.24%)

SYSUCC = Sun Yat-sen University Cancer Center; VMAT = volumetric modulated arc radiation therapy; IMRT = intensity-modulated radiation therapy; SBRT = stereotactic radiotherapy; TOMO = tomotherapy; 3D-CRT = three-dimensional conformal radiotherapy; N = numbers.

Table S3 Scores of EORTC QLQ-H&N35 (*n* = 437)

Scales/Items	Mean	SD	Median	IQR	N (%)
HN Pain	29.98	23.21	25	(8.33,41.67)	369 (84.44)
HN Swallowing	31.18	27.91	25	(8.33,50)	339 (77.57)
HN Senses problems	41.34	27.34	33.33	(16.67,50)	372 (85.13)
HN Speech problems	16.15	19.23	11.11	(0,22.22)	265 (60.64)
HN Trouble social eating	29.31	22.25	25	(8.33,41.67)	376 (86.04)
HN Trouble social contact	6.61	8.19	3.33	(0,10)	258 (59.04)
HN Less sexuality	37.8	34.9	33.33	(0,66.67)	290 (66.36)
HN Teeth	11.37	19.56	0	(0,33.33)	129 (29.52)
HN Opening mouth	16.09	21.72	0	(0,33.33)	181 (41.42)
HN Dry mouth	53.47	30.48	33.33	(33.33,66.67)	398 (91.08)
HN Sticky saliva	52.94	33.4	33.33	(33.33,66.67)	374 (85.58)
HN Coughing	21.89	24.76	33.33	(0,33.33)	232 (53.09)
HN Felt ill	25.55	28.39	33.33	(0,33.33)	241 (55.15)
HN Pain killers*	24.94	43.32	0	(0,0)	109 (24.94)
HN Nutritional supplements*	61.33	48.76	100	(0,100)	268 (61.33)
HN Feeding tube*	3.89	19.36	0	(0,0)	17 (3.89)
HN Weight loss*	81.46	38.9	100	(100,100)	356 (81.46)
HN Weight gain*	2.75	16.36	0	(0,0)	12 (2.75)
Overall QoL Score	35.7	14.67	35.8	(25.49, 45.18)	437 (100)

EORTC = European Organization for Research and Treatment of Cancer; QLQ = quality of life questionnaire; HN = head and neck; QoL = quality of life; SD = standard deviation; IQR = interquartile range; N = numbers; * binary (no/yes) items.

Table S4 Baseline characteristics of patients across different quality of life groups

Variable	Category	Total (%) (*n* = 437)	Poorer QoL (*n* = 240)	Better QoL (*n* = 197)	*P* value
**Age (year)**	Median (IQR)	47 (38, 55)	46 (37, 54)	49 (38, 57)	0.066
	Range	20-82	23-82	20-78	
**Gender**	Male	321 (73.46%)	166 (51.71%)	155 (48.29%)	0.025*
	Female	116 (26.54%)	74 (63.79%)	42 (36.21%)	
**Occupational status**	Employed	391 (89.47%)	215 (54.99%)	176 (45.01%)	0.934
	Unemployed	46 (10.53%)	25 (54.35%)	21 (45.65%)	
**Family annual income, CNY**	≥ 100,000	195 (44.62%)	110 (56.41%)	85 (43.59%)	0.574
	< 100,000	242 (55.38%)	130 (53.72%)	112 (46.28%)	
**Marital status**	Married	382 (87.41%)	207 (54.19%)	175 (45.81%)	0.418
	Single	55 (12.59%)	33 (60%)	22 (40%)	
**Smoking**	Active/Quit	184 (42.11%)	99 (53.8%)	85 (46.2%)	0.689
	Never	253 (57.89%)	141 (55.73%)	112 (44.27%)	
**Tumor category**	T1-2	44 (10.07%)	26 (59.09%)	18 (40.91%)	0.558
	T3-4	393 (89.93%)	214 (54.45%)	179 (45.55%)	
**Nodal category**	N0-1	188 (43.02%)	100 (53.19%)	88 (46.81%)	0.528
	N2-3	249 (56.98%)	140 (56.22%)	109 (43.78%)	
**AJCC 8th Stage**	I-II	25 (5.72%)	13 (5.42%)	12 (6.09%)	0.763
	III-IV	412 (94.28%)	227 (94.58%)	185 (93.91%)	
**EBV-DNA†**	Detectable	139 (31.81%)	177 (59.4%)	121 (40.6%)	0.006*
	Undetectable	298 (68.19%)	63 (45.32%)	76 (54.68%)	
**Cumulative fraction, Fr**	Median (IQR)	18 (11, 25)	22.5 (17, 28)	12 (6, 20)	< 0.001*
	Range	1-33	3-33	1-33	
**Cumulative dosage, cGy**	Median (IQR)	3816 (2237, 5300)	4876 (3601, 5936)	2544 (1272, 4240)	< 0.001*
	Range	212-7008	600-7008	212-7006	
**Replanning**	Yes	15 (3.43%)	9 (3.75)	6 (3.05%)	0.687
	No	422 (96.57%)	231 (96.25%)	191 (96.95%)	
**Treatment modality**	RT	25 (5.72%)	12 (48%)	13 (52%)	0.456
	CCRT	51 (11.67%)	33 (64.71%)	18 (35.29%)	
	SCRT	16 (3.66%)	9 (56.25%)	7 (43.75%)	
	IC+CCRT	345 (78.95%)	186 (53.91%)	159 (46.09%)	
**Targeted therapy**	Yes	248 (56.75%)	106 (56.08%)	83 (43.92%)	0.669
	No	189 (43.25%)	134 (54.03%)	114 (45.97%)	
**Immunotherapy**	Yes	362 (82.84%)	39 (52%)	36 (48%)	0.577
	No	75 (17.16%)	201 (55.52%)	161 (44.48%)	
**Body compositions**	Height (cm), Median (IQR)	165 (160, 170)	165 (159, 170)	166 (162, 170)	0.200
	Weight (kg), Median (IQR)	64 (56.1, 72)	63 (56.25, 72)	65 (56.1, 72)	0.908
	BMI (kg/m^2), Median (IQR)	23.44 (21.47, 25.63)	23.69 (21.54, 25.84)	23.21 (21.41, 25.38)	0.229
	SMI (cm^2/m^2),	47.37	47.375	47.37	0.394

	Median (IQR)	(41.24, 51.84)	(40.75, 51.16)	(42.16, 52.51)	
	LBM (kg),	45.99	45.875	46.08	0.328
	Median (IQR)	(37.82, 50.67)	(36.92, 50.54)	(38.83, 50.88)	
**Weight loss in the past 6 months**	< 5%	278 (63.62%)	51 (32.08%)	108 (67.92%)	< 0.001*
	≥ 5%	159 (36.38%)	189 (67.99%)	89 (32.01%)	
**Modified weight loss grading system, mWLGS**	Grade 0	12 (2.75%)	2 (0.83%)	10 (5.08%)	< 0.001*
	Grade 1	67 (15.33%)	16 (6.67%)	51 (25.89%)	
	Grade 2	112 (25.63%)	50 (20.83%)	62 (31.47%)	
	Grade 3	153 (35.01%)	102 (42.5%)	51 (25.89%)	
	Grade 4	93 (21.28%)	70 (29.17%)	23 (11.68%)	
**Malnutrition defined by mPG-SGA**	Total scores, Median (IQR)	11 (6.5, 14)	13 (10, 16)	7 (4, 11)	< 0.001*
	None/Well	6 (1.37%)	0 (0%)	6 (100%)	< 0.001*
	Mild	24 (5.49%)	2 (8.33%)	22 (91.67%)	
	Moderate	79 (18.08%)	16 (20.25%)	63 (79.75%)	
	Severe	328 (75.06%)	222 (67.68%)	106 (32.32%)	

QoL = quality of life; IQR = interquartile range; CNY = ChinaYuan; AJCC = American Joint Committee on Cancer staging system; EBV = Epstein-Barr Virus; DNA = DeoxyriboNucleic Acid; † Pre-radiotherapy EBV DNA copy numbers; Fr = fraction; cGy = centi-Gray; RT = radiotherapy; CCRT = concurrent radiotherapy; SCRT = sequential chemoradiotherapy; BMI = body mass index; SMI = skeletal muscular index; LBM = lean body mass. mPG-SGA = modified Patient-Generated Subjective Global Assessment; Well-nourished (0 point); Mild malnutrition (1-2 points); Moderate malnutrition (3-6 points); Severe malnutrition (≥ 7 points). * significant *P* value.

Table S5 Weight of each connection in the GLASSO network without clinical covariates

	A1	A2	A3	A4	A5	A6	A7	A8	A9	B1	B2	B3	B4	B5	C1	C2	C3	C4	Nutr	QoL
A1	0	0.12	0.21	0.17	0.09	0.22	0.04	0.04	0.08	0	0.03	-0.06	0	0	0	0	0.06	0	0	0.02
A2	0.12	0	0.2	0.01	0.05	0.08	0.1	0.15	0.01	0	0	0	0	0	0	0.07	0	0	0	0.05
A3	0.21	0.2	0	0.13	0.09	0	0.09	0.02	0.01	0	0	0	0.03	0	0.03	0.01	0.02	0.03	0	0
A4	0.17	0.01	0.13	0	0.16	0.09	0.1	0.06	0	0	0	0	0	0	0.06	0.08	0	0.02	0.01	0
A5	0.09	0.05	0.09	0.16	0	0.12	0	0.05	0.24	0.01	0	0	0	0.04	0.06	0	0.06	0.01	0.08	0
A6	0.22	0.08	0	0.09	0.12	0	0.01	0	0	0.05	0	0	0.07	0.03	0	0	0	0.02	0	0
A7	0.04	0.1	0.09	0.1	0	0.01	0	0	0.07	0	0.03	0	0.13	0	0.02	0.02	0	0.06	0	0
A8	0.04	0.15	0.02	0.06	0.05	0	0	0	0	0	0.05	0	0.17	0.09	0	0.05	0	0.02	0.02	0.09
A9	0.08	0.01	0.01	0	0.24	0	0.07	0	0	0	0.03	0.02	0	0	0.02	0.03	0	0.02	0.21	0
B1	0	0	0	0	0.01	0.05	0	0	0	0	0.57	0.08	0.1	0.05	0	0	0.08	0.05	0.04	0.24
B2	0.03	0	0	0	0	0	0.03	0.05	0.03	0.57	0	0	0	0.06	0	0.01	0	0	0.05	0.04
B3	-0.06	0	0	0	0	0	0	0	0.02	0.08	0	0	0.17	0.11	0	0	0.09	0	0.12	0.13
B4	0	0	0.03	0	0	0.07	0.13	0.17	0	0.1	0	0.17	0	0	0	0	0	0.05	0.05	0.06
B5	0	0	0	0	0.04	0.03	0	0.09	0	0.05	0.06	0.11	0	0	0.01	0	0.25	0.07	0.04	0.09
C1	0	0	0.03	0.06	0.06	0	0.02	0	0.02	0	0	0	0	0.01	0	0.6	0.24	0	0.04	0
C2	0	0.07	0.01	0.08	0	0	0.02	0.05	0.03	0	0.01	0	0	0	0.6	0	0	0.08	0.09	0
C3	0.06	0	0.02	0	0.06	0	0	0	0	0.08	0	0.09	0	0.25	0.24	0	0	0.01	0.16	0.01
C4	0	0	0.03	0.02	0.01	0.02	0.06	0.02	0.02	0.05	0	0	0.05	0.07	0	0.08	0.01	0	0.11	0.04
Nutr	0	0	0	0.01	0.08	0	0	0.02	0.21	0.04	0.05	0.12	0.05	0.04	0.04	0.09	0.16	0.11	0	0.14
QoL	0.02	0.05	0	0	0	0	0	0.09	0	0.24	0.04	0.13	0.06	0.09	0	0	0.01	0.04	0.14	0

A1: Anxiety; A2: Shortness of breath; A3: Palpitations; A4: Dizziness; A5: Fatigue; A6: Insomnia; A7: Abdominal pain; A8: Cough; A9: Immobility; B1: Dysphagia; B2: Mucositis or sore throat; B3: Hypogeusia; B4: Radiation-induced skin injury; B5: Xerostomia; C1: Nausea; C2: Vomiting; C3: Anorexia; C4: Constipation; Nutr: Malnutrition; QoL: Quality of life.

Table S6 Spearman correlation coefficient of PRO-CTCAE and malnutrition

	r	*P*
Decreased appetite	0.571	< 0.001
Dysphagia	0.531	< 0.001
Immobility	0.524	< 0.001
Nausea	0.517	< 0.001
Vomiting	0.516	< 0.001
Fatigue	0.498	< 0.001
Mouth/throat sores	0.481	< 0.001
Xerostomia	0.473	< 0.001
Taste changes	0.468	< 0.001
Radiation skin reaction	0.417	< 0.001
Constipation	0.413	< 0.001
Cough	0.400	< 0.001
Dizziness	0.399	< 0.001
Anxiety	0.366	< 0.001
Palpitations	0.359	< 0.001
Shortness of breath	0.346	< 0.001
Abdominal pain	0.326	< 0.001
Insomnia	0.307	< 0.001

r: Spearman rank correlation coefficients.

Table S7 Weight of each connection in the GLASSO network with clinical covariates

	A1	A2	A3	A4	A5	A6	A7	A8	A9	B1	B2	B3	B4	B5	C1	C2	C3	C4	Nutr	QoL	D1	D2	D3	D4
A1	0	0.12	0.21	0.17	0.09	0.21	0.04	0.04	0.08	0	0.03	0	0	0	0	0	0.06	0	0	0.01	-0.04	-0.07	0	0
A2	0.12	0	0.19	0.02	0.05	0.08	0.1	0.15	0.02	0	0	0	0	0	0	0.06	0	0	0	0.05	0	0	0	0
A3	0.21	0.19	0	0.13	0.09	0	0.09	0.02	0.01	0	0	0	0.03	0	0.03	0.01	0.02	0.03	0	0	0	0	0	0
A4	0.17	0.02	0.13	0	0.15	0.09	0.1	0.07	0	0	0	0	0	0	0.07	0.08	0	0.03	0.02	0	-0.05	-0.02	0	0
A5	0.09	0.05	0.09	0.15	0	0.12	0	0.05	0.23	0.01	0	0	0	0.04	0.06	0	0.06	0.01	0.08	0	0	0	0	0
A6	0.21	0.08	0	0.09	0.12	0	0.01	0	0	0.06	0	0	0.06	0.02	0	0	0	0.02	0	0	0	0	0	0
A7	0.04	0.1	0.09	0.1	0	0.01	0	0	0.07	0	0.03	0	0.12	0	0.02	0.02	0	0.05	0	0	0	0	0	0
A8	0.04	0.15	0.02	0.07	0.05	0	0	0	0	0	0.04	0	0.16	0.09	0	0.05	0	0.02	0.02	0.09	0.03	0	0	0
A9	0.08	0.02	0.01	0	0.23	0	0.07	0	0	0	0.04	0.01	0	0	0.02	0.03	0	0.02	0.21	0	0	0	0	0
B1	0	0	0	0	0.01	0.06	0	0	0	0	0.54	0.05	0.09	0.05	0	0	0.09	0.05	0.04	0.23	0.04	0	0	0
B2	0.03	0	0	0	0	0	0.03	0.04	0.04	0.54	0	0	0	0.06	0	0.01	0	0	0.04	0.04	0.1	0	0	0
B3	0	0	0	0	0	0	0	0	0.01	0.05	0	0	0.11	0.1	0	0	0.08	0	0.09	0.1	0.21	0	0.1	0
B4	0	0	0.03	0	0	0.06	0.12	0.16	0	0.09	0	0.11	0	0	0	0	0	0.04	0.04	0.05	0.17	0	0	0
B5	0	0	0	0	0.04	0.02	0	0.09	0	0.05	0.06	0.1	0	0	0.01	0	0.24	0.06	0.04	0.09	0	0	0	0
C1	0	0	0.03	0.07	0.06	0	0.02	0	0.02	0	0	0	0	0.01	0	0.58	0.23	0	0.04	0	0	0	0	0
C2	0	0.06	0.01	0.08	0	0	0.02	0.05	0.03	0	0.01	0	0	0	0.58	0	0.01	0.08	0.09	0	0	-0.08	0.02	0
C3	0.06	0	0.02	0	0.06	0	0	0	0	0.09	0	0.08	0	0.24	0.23	0.01	0	0.01	0.15	0.02	0	0	0.01	0
C4	0	0	0.03	0.03	0.01	0.02	0.05	0.02	0.02	0.05	0	0	0.04	0.06	0	0.08	0.01	0	0.09	0.03	0.01	0	0.07	0
Nutr	0	0	0	0.02	0.08	0	0	0.02	0.21	0.04	0.04	0.09	0.04	0.04	0.04	0.09	0.15	0.09	0	0.12	0.03	0	0.09	0
QoL	0.01	0.05	0	0	0	0	0	0.09	0	0.23	0.04	0.1	0.05	0.09	0	0	0.02	0.03	0.12	0	0.04	0	0.1	0.05
D1	-0.04	0	0	-0.05	0	0	0	0.03	0	0.04	0.1	0.21	0.17	0	0	0	0	0.01	0.03	0.04	0	0	0.14	0
D2	-0.07	0	0	-0.02	0	0	0	0	0	0	0	0	0	0	0	-0.08	0	0	0	0	0	0	0	0
D3	0	0	0	0	0	0	0	0	0	0	0	0.1	0	0	0	0.02	0.01	0.07	0.09	0.1	0.14	0	0	0
D4	0	0	0	0	0	0	0	0	0	0	0	0	0	0	0	0	0	0	0	0.05	0	0	0	0

A1: Anxiety; A2: Shortness of breath; A3: Palpitations; A4: Dizziness; A5: Fatigue; A6: Insomnia; A7: Abdominal pain; A8: Cough; A9: Immobility; B1: Dysphagia; B2: Mucositis or sore throat; B3: Hypogeusia; B4: Radiation-induced skin injury; B5: Xerostomia; C1: Nausea; C2: Vomiting; C3: Anorexia; C4: Constipation; Nutr: Malnutrition; QoL: Quality of life; D1: Radiotherapy phase; D2: Gender; D3: Modified weight loss grading system; D4: Pre-radiotherapy EBV DNA copy numbers.

Table S8 Multivariate logistic regression analysis of quality of life adding symptoms as continuous variables

Items	OR (95% CI)	β	*P*
mWLGS Grade 3-4*	2.712 (1.518, 4.845)	0.998	0.001
Head-neck SC†	4.513 (2.333, 8.73)	1.507	< 0.001
mPG-SGA total scores†	1.487 (1.004, 2.201)	0.397	0.048
Dysphagia†	1.83 (1.076, 3.114)	0.604	0.026
Shortness of breath†	2.109 (1.374, 3.237)	0.746	0.001
Cough†	1.448 (1.001, 2.095)	0.37	0.049

mWLGS = modified weight loss grading system; * as a binary variable with reference of grade 0-2 for the largest $R^2 = 0.657$; † as continuous variables after normalizations; SC = symptom cluster; mPG-SGA = modified Patient-Generated Subjective Global Assessment; OR= odds ratio; CI = confidence interval.

Table S9 Exploratory factor analysis in the early and late radiotherapy phases

Symptom cluster	Symptoms	Factor loading of early RT			Factor loading of late RT		
		1	2	3	1	2	3
Cluster 1: General symptom cluster	A1: Anxiety	0.708*	0.29	-0.445	0.83*	0.364	-0.191
	A2: Shortness of breath	0.768*	0.195	-0.34	0.732*	0.302	-0.249
	A3: Palpitations	0.728*	0.233	-0.372	0.736*	0.382	-0.306
	A4: Dizziness	0.565	0.24	-0.642*	0.752*	0.396	-0.225
	A5: Fatigue	0.648*	0.459	-0.565	0.714*	0.459	-0.365
	A6: Insomnia	0.618*	0.266	-0.279	0.618*	0.357	0.037
	A7: Abdominal pain	0.434	0.237	-0.543*	0.643*	0.276	-0.094
	A8: Cough	0.689*	0.372	-0.253	0.609*	0.389	0.046
	A9: Immobility	0.568*	0.386	-0.495	0.505*	0.363	-0.408
Cluster 2: Head-neck symptom cluster	B1: Dysphagia	0.344	0.871*	-0.288	0.569	0.763*	0.093
	B2: Mouth/throat sores	0.265	0.846*	-0.276	0.548	0.633*	0.162
	B3: Taste changes	0.18	0.637*	-0.294	0.125	0.607*	-0.097
	B4: Radiation skin reaction	0.396	0.574*	-0.327	0.567*	0.382	0.23
	B5: Xerostomia	0.351	0.753*	-0.421	0.44	0.678*	-0.194
Cluster 3: Gastrointestinal symptom cluster	C1: Nausea	0.418	0.393	-0.884*	0.571	0.534	-0.717*
	C2: Vomiting	0.431	0.361	-0.859*	0.553	0.532	-0.652*
	C3: Decreased appetite	0.406	0.649	-0.68*	0.458	0.706*	-0.439
	C4: Constipation	0.234	0.291	-0.644*	0.396	0.59*	-0.114
Initial Eigenvalues		6.69	1.797	1.274	7.238	1.268	1.142
Cumulative %		37.17	47.15	54.23	40.21	47.25	53.60
Cronbach's α		0.819	0.805	0.821	0.871	0.714	0.833

* Loadings for the factor indicated. Underlined numbers indicate a change in cluster assignment compared to the whole participant set. RT = Radiotherapy. Early RT: < 18 fractions. Late RT: ≥ 18 fractions.

Table S10 Prevalence of acute symptoms in the previous study compared with our survey

Symptoms in previous study†	N%	Corresponding items in our study	N%
Problem with tasting food	96.9	Taste changes	96.2
Having a dry mouth	97.7	Xerostomia	95.44
Difficulty swallowing/chewing	98.5	Dysphagia	88.59
Lack of appetite	95.4	Decreased appetite	94.3
Mouth/throat sores	96.9	Mouth/throat sores	89.73
Nausea	91.5	Nausea	86.31
Disturbed sleep	94.5	Insomnia	63.12
Vomiting	81.5	Vomiting	73
Fatigue	93.8	HN Fatigue	86.69
Feelings of being distressed	86	Anxiety	56.65
Pain	83.8	HN Pain	95.44
Difficulty with voice/speech	85.4	HN Speech problems	72.62
Constipation	76.9	Constipation	68.82
Problem with teeth or gums	82.3	HN Teeth	35.74
Choking/coughing	73.8	Cough	62.36
Skin pain/burning/rash	76.7	Radiation skin reaction	68.06
Shortness of breath	67.7	Shortness of breath	33.46
General activity	69.2	Immobility	70.72

† Evaluated by the Chinese version of the M. D. Anderson Symptom Inventory-Head and Neck Module (MDASI-HN-C) and the Chinese version of the Functional Assessment of Cancer Therapy-Head and Neck Scale (FACT-H&N-C)[2]. HN: items evaluated by EORTC QLQ-H&N35.

2 Xiao W, Chan CWH, Fan Y, et al. Symptom clusters in patients with nasopharyngeal carcinoma during radiotherapy. European journal of oncology nursing: the official journal of European Oncology Nursing Society. Jun 2017; 28:7-13. DOI: 10.1016/j.ejon.2017.02.004

Table S11 Radiotherapy fractions of patients who had received replanning

Patient labels	Fractions of first replanning	Fractions during our survey
1	11	20
2	22	22
3	22	32
4	24	28
5	24	32
6	25	25
7	26	32
8	26	31
9	26	32
10	26	32
11	27	27
12	27	32
13	28	33
14	28	28
15	29	30

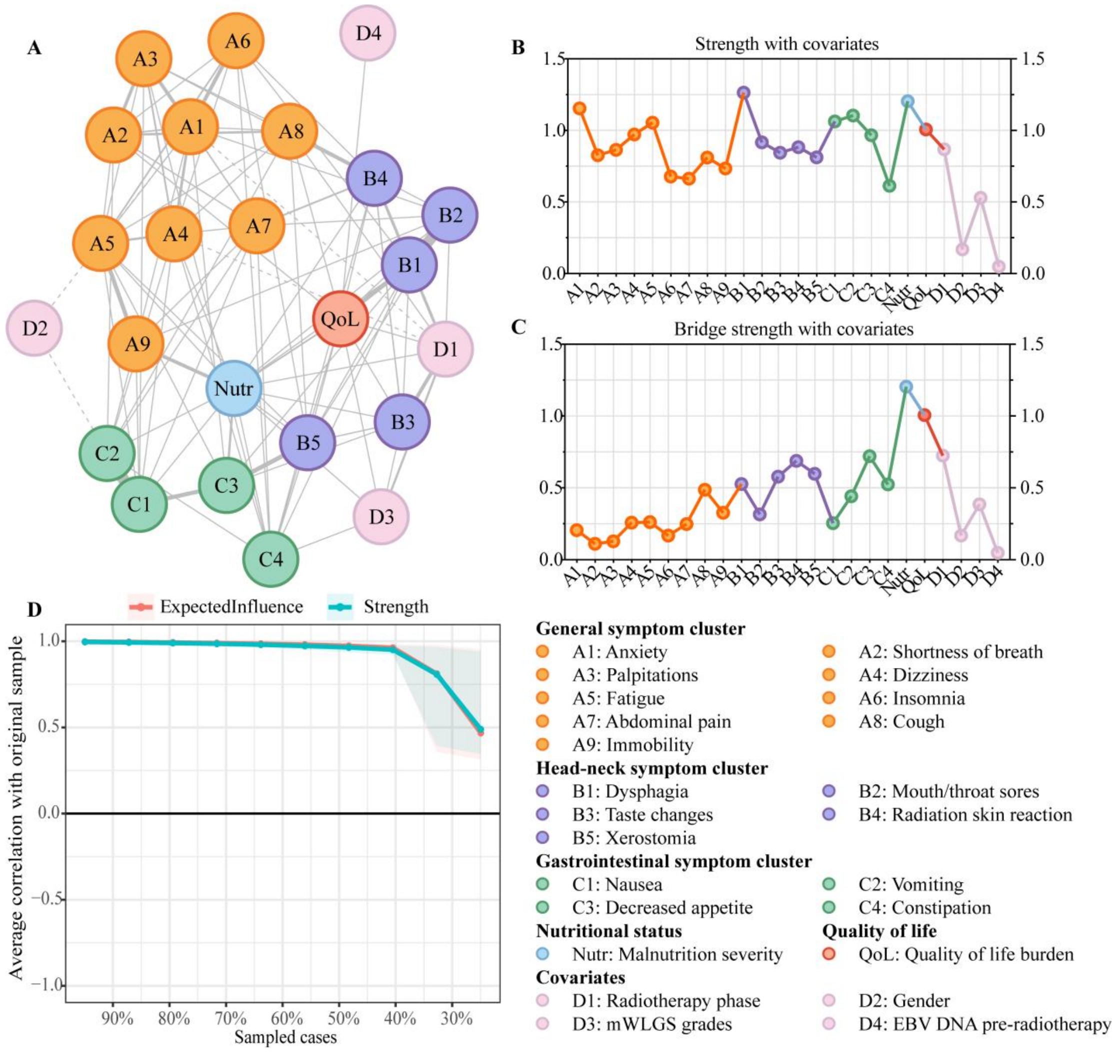

Fig.S1 Visual representation of the network after controlling for covariates

(A) Network model with nodes corresponding to symptoms. Solid grey lines denote positive correlations, and the dashed lines represent negative correlations. The length and thickness of the edges signify correlation strength.

(B-C) Centrality indices and bridging indices of nodes in the network. The x-axis marks the nodes of the network. The y-axis shows the z-score normalized value of these indices. Dot colors align with network (A)'s scheme.

(D) Centrality index stability evaluated by case dropping subset bootstrap. The x-axis represents the percentage of original cases sampled per step. The y-axis represents the correlation between original and post-bootstrapping re-estimated networks' centrality indices. Lines plot the estimated correlation coefficients at each step, with shaded areas denoting 95% confidence intervals.

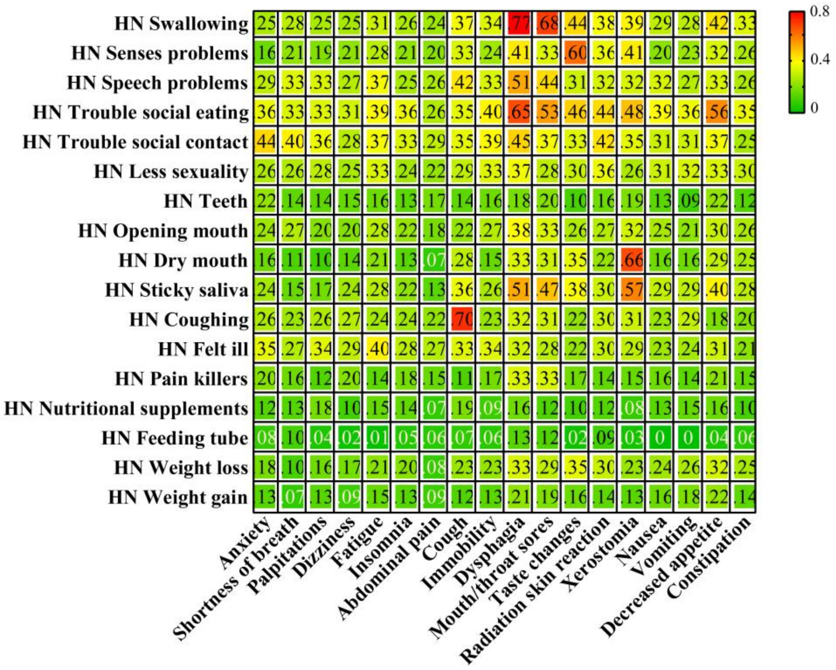

Fig.S2 Correlation matrix (Spearman, r) of the PRO-CTCAE and EORTC QLQ-H&N35 items

The x-axis displays the symptoms of PRO-CTCAE. The y-axis shows the normalized value of EORTC QLQ-H&N35 items. Numbers within the matrix represent the Spearman correlation coefficients, specifically the digits following the decimal point. Black font signifies a statistically significant correlation ($P < 0.05$), whereas white font indicates not. The background color of each cell in the matrix indicates the strength of the correlation: the closer the color is to red, the stronger the correlation is; conversely, the closer to green, the weaker the correlation is.

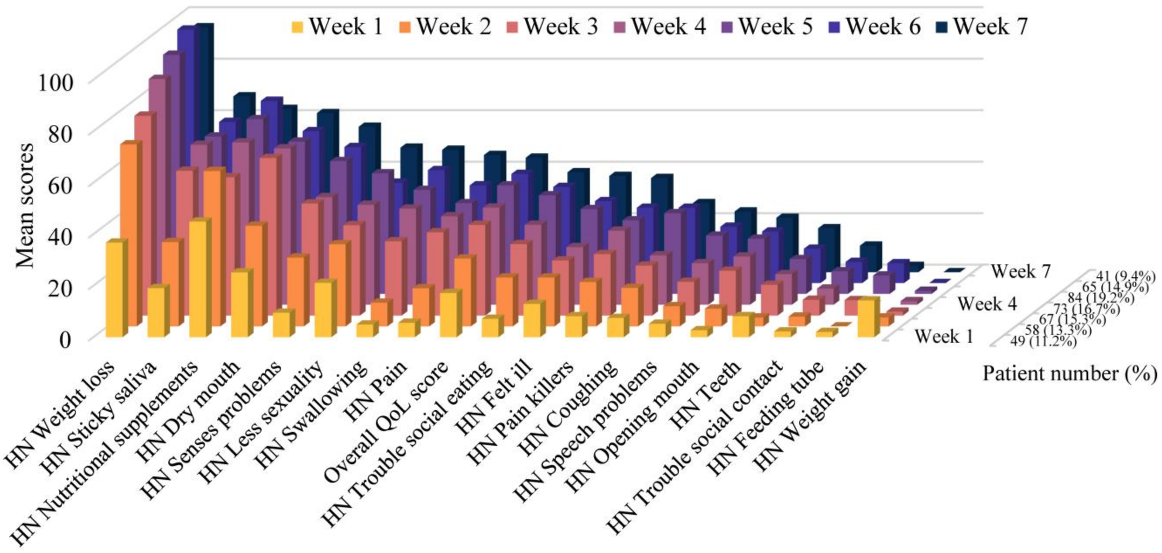

Fig.S3 Mean scores of EORTC QLQ-H&N35 scales during weeks 1-7 of radiotherapy

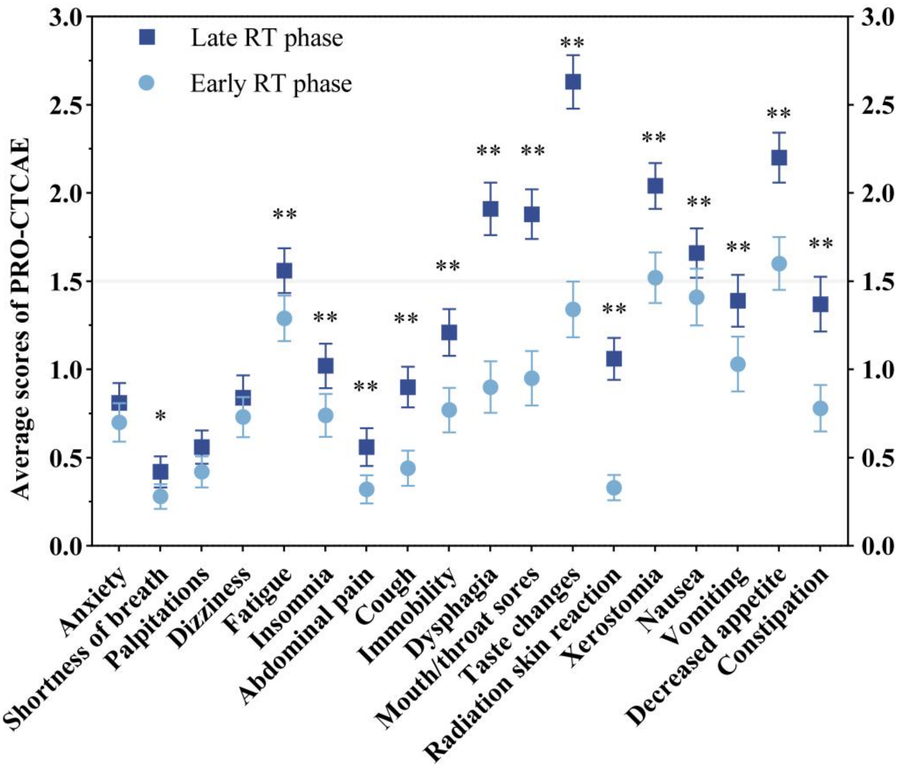

Fig.S4 Symptom severity distribution of PRO-CTCAE in the early and late radiotherapy phases

PRO-CTCAE = Patient-reported outcome version of the Common Terminology Criteria for Adverse Events; RT = radiotherapy. * $P < 0.05$; ** $P < 0.01$. Data are expressed as mean ± SEM in the plotting.

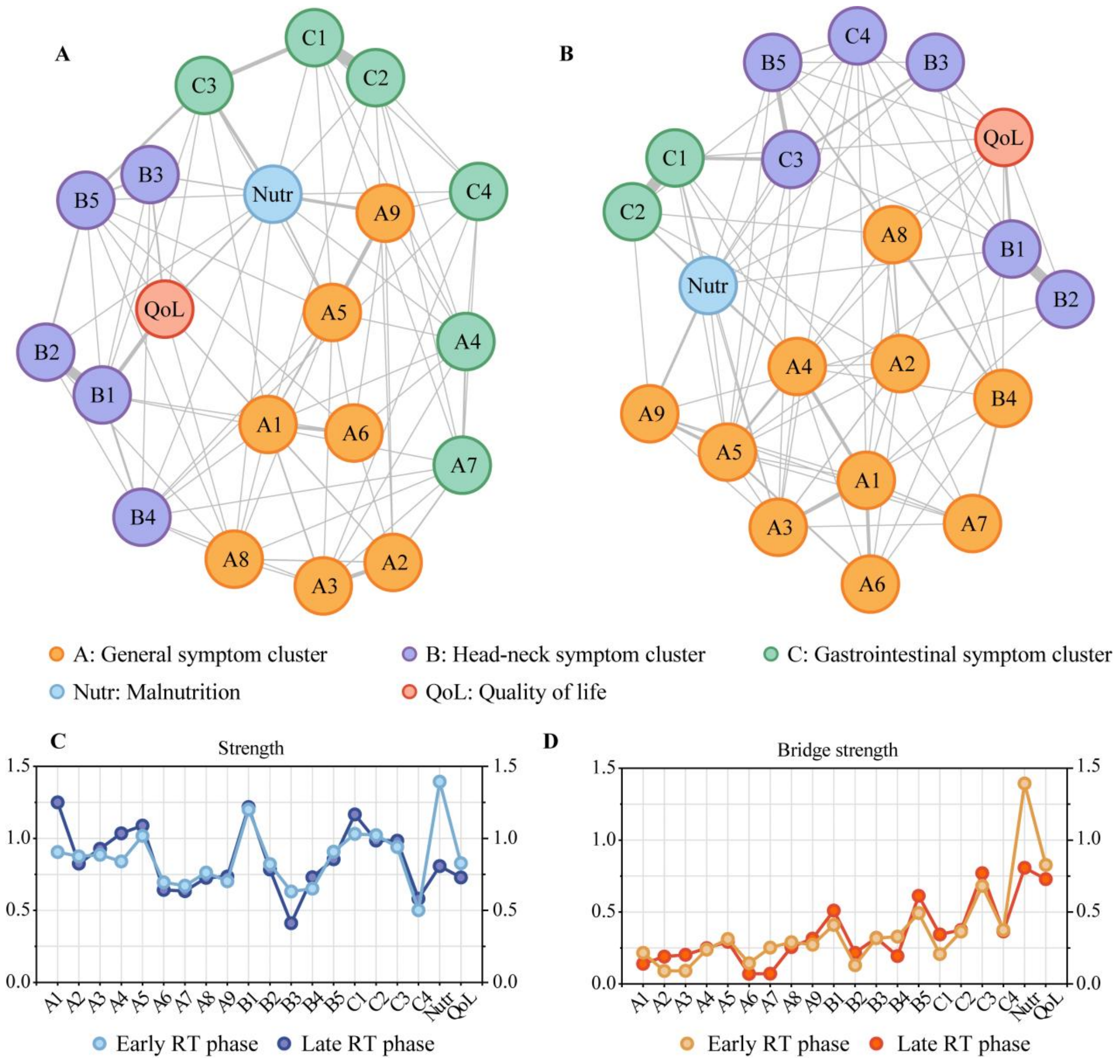

Fig.S5 Estimated network model for the association between symptom clusters, malnutrition, and QoL in the early and late radiotherapy phases

(A-B) Network model of early RT phase (A) and late RT phase (B) with nodes corresponding to symptoms. Solid grey lines denote positive correlations, and the dashed lines represent negative correlations. The length and thickness of the edges signify correlation strength.

(C-D) Centrality indices and bridging indices of nodes in the network. The x-axis marks the nodes of the network. The y-axis shows the z-score normalized value of these indices. Dot lighter colors represent the early RT phase, while the darker color represents the late RT phase.

A1: Anxiety; A2: Shortness of breath; A3: Palpitations; A4: Dizziness; A5: Fatigue; A6: Insomnia; A7: Abdominal pain; A8: Cough; A9: Immobility; B1: Dysphagia; B2: Mucositis or sore throat; B3: Hypogeusia; B4: Radiation-induced skin injury; B5: Xerostomia; C1: Nausea; C2: Vomiting; C3: Anorexia; C4: Constipation.

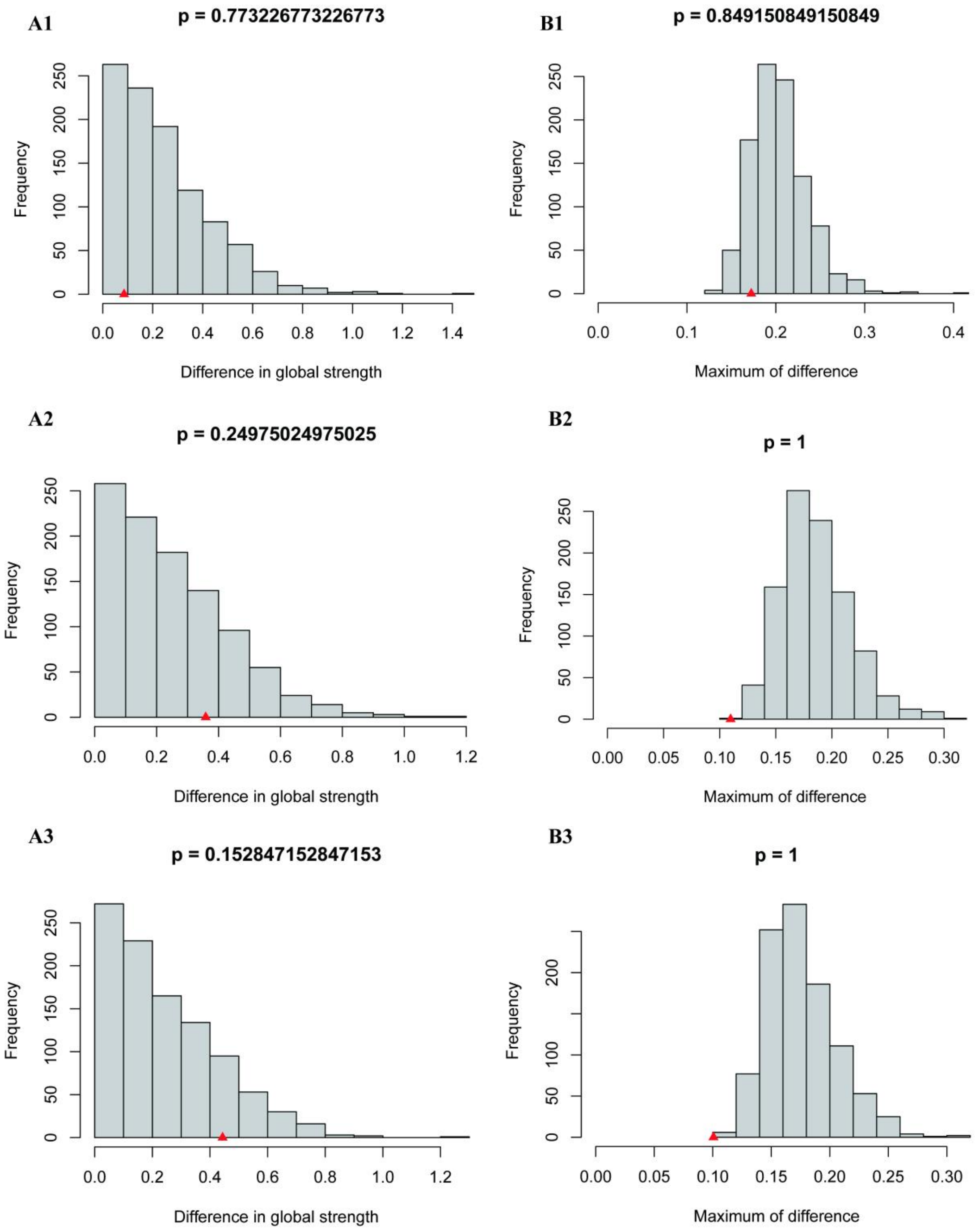

Fig.S6 Network strength invariance test and structure invariance test

Figures A1-A3 illustrate the findings from the network strength invariance analysis, comparing Network_early with Network_late (A1), Network_early with the entire population network (A2), and Network_late with the entire population network (A3). Correspondingly, B1-B3 depict the network structure invariance analysis results for the same pairs. Network_early means the network of the early RT phase (< 18 fractions), while network_late refers to the network of the late RT phase (≥ 18 fractions). The red triangle highlights the divergence between the original networks, and the histogram represents the bootstrapped estimates of the differences in network structure and overall strength.

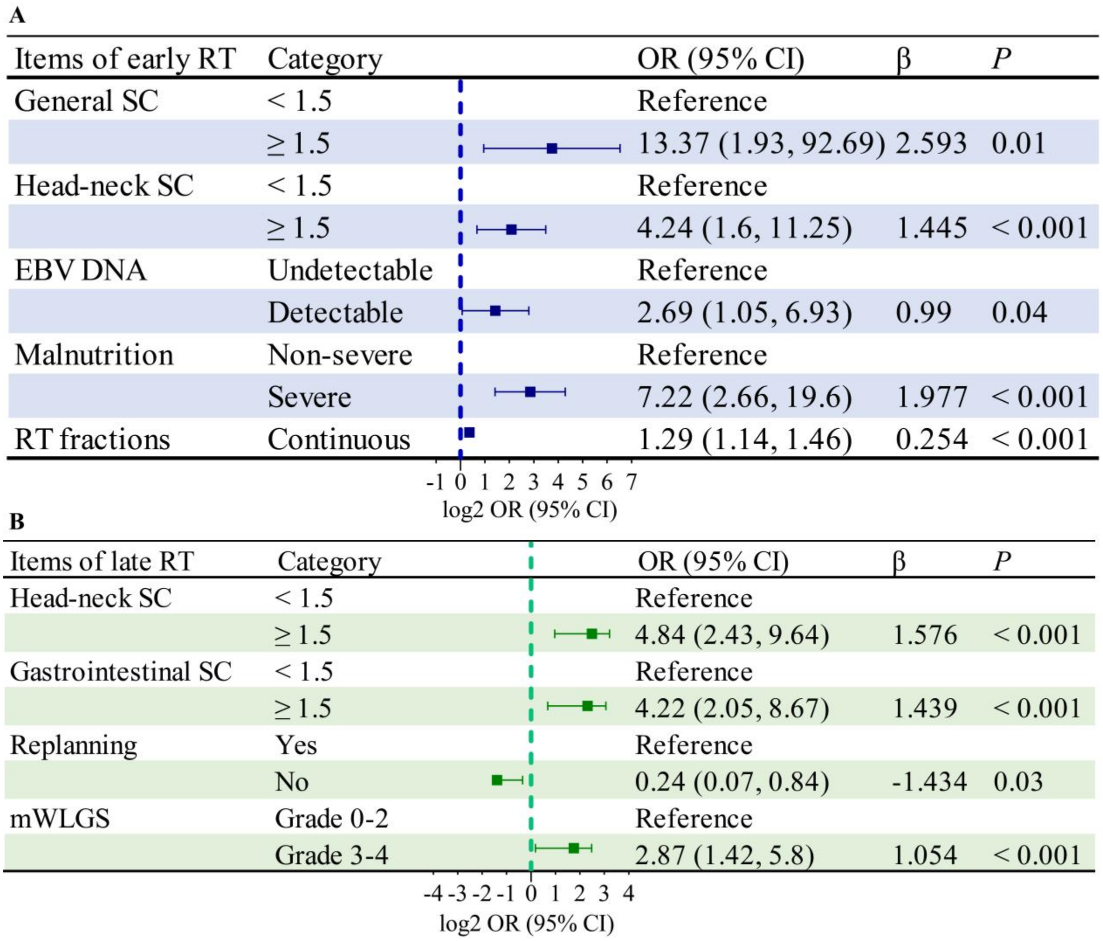

A

Items of early RT	Category	OR (95% CI)	β	*P*
General SC	< 1.5	Reference		
	≥ 1.5	13.37 (1.93, 92.69)	2.593	0.01
Head-neck SC	< 1.5	Reference		
	≥ 1.5	4.24 (1.6, 11.25)	1.445	< 0.001
EBV DNA	Undetectable	Reference		
	Detectable	2.69 (1.05, 6.93)	0.99	0.04
Malnutrition	Non-severe	Reference		
	Severe	7.22 (2.66, 19.6)	1.977	< 0.001
RT fractions	Continuous	1.29 (1.14, 1.46)	0.254	< 0.001

B

Items of late RT	Category	OR (95% CI)	β	*P*
Head-neck SC	< 1.5	Reference		
	≥ 1.5	4.84 (2.43, 9.64)	1.576	< 0.001
Gastrointestinal SC	< 1.5	Reference		
	≥ 1.5	4.22 (2.05, 8.67)	1.439	< 0.001
Replanning	Yes	Reference		
	No	0.24 (0.07, 0.84)	-1.434	0.03
mWLGS	Grade 0-2	Reference		
	Grade 3-4	2.87 (1.42, 5.8)	1.054	< 0.001

Fig.S7 Multivariate logistic regression analysis of quality of life in the early and late radiotherapy phases

(A) Significant items for the binary overall quality of life in the early RT phase (< 18 fractions). (B) Significant items for the binary overall quality of life in the late RT phase (≥ 18 fractions). RT = radiotherapy; SC = symptom cluster; EBV = Epstein-Barr Virus; DNA = DeoxyriboNucleic Acid; mWLGS = modified weight loss grading system; OR = odds ratio; CI = confidence interval.
